# Giving away some of their powers! Towards learner agency in digital assessment and feedback

**DOI:** 10.1186/s41039-021-00168-6

**Published:** 2021-07-07

**Authors:** Diogo Casanova, Graham Alsop, Isabel Huet

**Affiliations:** 1 Universidade Aberta & CIDTFF, Rua da Escola Politécnica 141, 1250-100 Lisbon, Portugal; 2 School of Computer Science and Mathematics, Kingston University London, Kingston, UK; 3 Departamento de Educação e Ensino a Distância, Universidade Aberta and CIDTFF, Lisboa, Portugal

**Keywords:** Human-computer interface, Pedagogical issues, Post-secondary education, Information literacy, Architectures for educational technology system

## Abstract

Digital assessment and feedback have been a growing area of research and practice in the past decade in higher education. Within this theme, research has been published highlighting the importance of learner agency in the assessment and feedback process as a way to develop assessment literacy in contrast with the existing lecturer-led approach. In this research, we aimed to find out whether lecturers are willing to let go of some of the power they currently have in the digital assessment and feedback process and how they see opportunities for agency being developed in the digital assessment and feedback systems. We collected data from 10 sandpits with 58 lecturers in which, using a storytelling technique and one mock-up of a digital assessment and feedback system, we discussed and critiqued an assessment scenario intending to collect perceptions about digital assessment and feedback and the constraints felt by lecturers in their assessment practice. Based on these perceptions, we identify recommendations that may improve digital assessment and feedback systems and practices. We discuss the data and the recommendations based on three clusters of themes: (i) preparation for the assessment, (ii) formative feedback and (iii) feedback post-submission.

**Supplementary Information:**

The online version contains supplementary material available at 10.1186/s41039-021-00168-6.

## Introduction

Digital assessment and feedback are areas of increasing interest in the higher education (HE) sector, particularly at a time when there is increased demand for more blended and distance learning environments, motivated by Covid-19, the globalisation of HE and the massification of the HE market. In this research, we will be dwelling on a large area of assessment in HE—the Electronic Management of Assessment (EMA) which provides a framework for the life cycle of the assessment and feedback process (Bausili, 2018). Digital technologies have been seen as tools to promote more efficiency and access to the assessment and feedback process, not just because of the cost and efficiency savings in transcribing grades and the reduction of the risk of errors, but also to enable learners to have faster access to grades and feedback (Bausili, 2018; Debuse & Lawley, 2016; Fulda, 2006).

Digital assessment and feedback are gradually changing how assessment and feedback are done: before digital assessment and feedback systems (DAFS) were developed, coursework was taken either on paper or a disc to the administrator’s office or to the lecturer. Feedback was then given in writing, was frequently misunderstood by the learner and was stored somewhere on a shelf and forgotten about. Feedback was unidirectional, as learners would read the feedback provided but seldomly act on it. With the wide use of DAFS, we now have at our disposal more effective, accessible and transparent practices, but rather than embracing its potential to store and reuse feedback, DAFS providers seem to be mimicking the ‘pen and paper’ experience, thereby influencing how lecturers engage with this process (Casanova et al., 2020). Influenced by how DAFS are designed, many universities appear to favour a transmission model of assessment and feedback by positioning learners as passive receivers of information about their work and not as active agents of their assessments (Beaumont, O’Doherty, & Shannon, 2011; Boud & Falchikov, 2006; Nieminen & Tuohilampi, 2020). This is still rooted in a traditional model of assessment where feedback is used not to improve learning but to justify a grade (Bailey & Garner, 2010).

However, there is growing evidence supporting the value of feedback for learning; the view that feedback on learner performance is one of the most influential and effective learning paradigms (Hattie & Timperley, 2007; Hepplestone, Holden, Irwin, Parkin, & Thorpe, 2011) and that socio-constructivism representations of the feedback process are more appropriate for learning than cognitivist transmission-oriented models (Winstone, Bourne, Medland, Niculescu, & Rees, 2020). Equally, there is growing evidence that the more learners actively engage with feedback the more they learn from it and the more effective it becomes (Beaumont et al., 2011; Boud & Falchikov, 2006; Carless & Boud, 2018; Winstone & Boud, 2019). Different authors have looked at the lens of learner agency to discuss learners’ engagement with assessment and feedback and as a way to improve assessment and feedback literacy (Nieminen & Tuohilampi, 2020; Winstone et al., 2020; Winstone, Nash, Parker, & Rowntree, 2017). Although some research has been trying to evaluate lecturers’ perceptions of quality feedback (Dawson et al., 2019; Mimirinis, 2019), little research has looked at how lecturers see learners’ active role in the digital assessment and feedback process and how DAFS can be used to promote this active role. In this paper, we want to look at lecturers’ perceptions of learner agency in assessment. Specifically, this study aims at responding to the question: how can lecturers and universities improve digital assessment and feedback systems (DAFS) that lead to an increase in learner agency in assessment and feedback?

The concept of agency leads to discussions about power balance, particularly in an area that has been mainly controlled by lecturers (Nieminen & Hilppö, 2020). Without lecturers giving away some of the power they currently possess in the assessment and feedback process, real learner agency will be difficult to achieve. With this premise in mind, we wanted to understand whether lecturers were willing to give away some of their power in the assessment process and how learner agency in assessment can be promoted in DAFS.

The paper starts with a literature review about digital assessment and feedback particularly focusing on how DAFS have been used to support the assessment and feedback process. We then present a conceptual overview of learner agency in HE and discuss the notion of power in assessment and feedback. This is considered instrumental as it provides a framework that informs both the research methods and the data analysis. Then, the research methods and data findings are presented. The paper finishes by providing recommendations to DAFS developers, lecturers and HE institutions on how learner agency can be incorporated into digital assessment and feedback practices.

## Literature review

Virtual learning environments (VLEs) and electronic marking tools, such as *Turnitin*, have been having a significant impact in assessment and feedback practice as well as institutional and national policies (ElShaer, Casanova, Freestone & Calabrese, 2020; Bausili, 2018). There is an argument that assessment and feedback practice is shaped by the way learning technologies are designed and the features available. Indeed, many authors discuss how technology design and its features may shape or influence teaching practices (Farrell & Rushby, 2016; Selwyn, 2013; Winstone et al., 2020) and how in turn influence student’s perception of their role in assessment (ElShaer, Casanova, Freestone & Calabrese, 2020; Henderson, Selwyn, & Aston, 2017).

### The role of DAFS in shaping the assessment and feedback process

Next, we explore how DAFS are being used to support the assessment and feedback process, looking particularly at how they can enhance a learner-led approach. A wide range of research conducted in the UK and Australasia has been exploring the impact of DAFS on learners’ engagement with the assessment process, particularly their engagement with feedback (Debuse & Lawley, 2016; Hepplestone et al., 2011; Parkin, Hepplestone, Holden, Irwin, & Thorpe, 2012; Winstone, 2019; Winstone et al., 2020; Zimbardi et al., 2017). Some of these studies specifically report on the positive outcomes of developing DAFS, targeting the improvement of particular areas of the feedback process to address pedagogical needs (Debuse & Lawley, 2016; Parkin et al., 2012; Winstone, 2019; Zimbardi et al., 2017).

Zimbardi et al. (2017), for example, designed a tool that supported the creation of different assignments, built from earlier assignments, with the links in a sequence made explicitly (e.g. with overlapping features) so that learners were more likely to draw on the feedback they received from preceding tasks (Zimbardi et al., 2017). The authors concluded that learners engaged with feedback as they saw relevance in the feedback received. They also found that this engagement led to a significant impact on performance, particularly for those learners that had engaged earlier with the feedback. Conversely, those who had limited or no interaction with their feedback did not improve to the same extent. Parkin et al. (2012) added that the storage of digital feedback increases the likelihood of learners revisiting their feedback and feeding it forward into future assignments. Winstone (2019) went a little bit further by developing a new system, which she named the Feedback Engagement and Tracking System (FEATS). The system had the potential to store all feedback received by the learners during their course and to track the impact of the feedback by allowing the learner to synthesise multiple feedback exchanges, visualise their key strengths and areas for development and record and monitor actions based on feedback received. This is an example of providing agency to the learner in the feedback process and of developing assessment literacy. Nevertheless, one could discuss whether all learners are capable of autonomously developing such skills. That is perhaps why Winstone (2019) argues that the process needs to be explicitly visible to the learner and to the lecturer.

Debuse and Lawley (2016) discussed the development of SMI (SuperMarkIt). This stand-alone system was developed to improve the quality of learner feedback whilst reducing lecturers’ workload. It focuses on very simple feedback dialogue boxes informed by a grading sheet with criteria. One can argue that this system was built to allow efficiency and consistency in the assessment and feedback process rather than to encourage learners to engage with the feedback received. In fact, learners were more positive about elements such as the legibility, timeliness and specificity of the feedback rather than whether it was constructive, varied in content or easy to understand. It is important to note that SMI enabled lecturers to grade swiftly and consistently provided that the assessment rubric was created (Debuse & Lawley, 2016). The work by Debuse and Lawley (2016) highlights the tension between efficiency in assessment and feedback and a student-led approach. A similar finding is shared by Penuel, Roschelle, and Shechtman (2007) who suggest that educators want to find solutions for their own perceived dilemmas rather than explore new and improved pedagogical approaches to engage students in formative feedback.

### Learner agency and power balance in assessment

There is no broad consensus on the definition of learner agency although the majority of authors refer to ownership or a sense of ownership of the learning process and the ability to make decisions or have a voice (Charteris & Smardon, 2018; Matusov, von Duyke, & Kayumova, 2016). Agency is not a new concept; it has been discussed as part of libertarian and neoliberal views of society where the individual is empowered to make choices about their own life (Matusov et al., 2016), and it has been linked in education to theories like self-determination (Ryan & Deci, 2000), the development of a ‘growth mindset’ (Dweck, 2008) and self-regulation (Bandura, 2001; Martin, 2004). The humanistic movement frames the concept of power as a product of agency with which individuals are endowed naturally. This power can be deployed or taken back (Charteris & Smardon, 2018).

For this research, we look at agency as ‘new material agency’, as defined by Charteris and Smardon (2018). Agency is a dynamic process that is generated through a range of elements within the educational setting. Agency is co-produced by the individual when it relates to objects and humans rather than possessing an ‘ontological existence that is devoid of agency’ (Charteris & Smardon, 2018, p. 61). By making explicit elements of agency when implementing a new generation of learning environments, the authors suggest that it ‘may strengthen and enhance learners positioning about their own learning’ (Charteris & Smardon, 2018, p. 55). A similar approach to learner agency is provided by Matusov et al. (2016), who discuss the existence of an emergent process that brings something new, innovative and creative to the learning process; those changes in context may place different demands on learners, which in turn develop different competencies. Charteris and Smardon (2018) add that making explicit learner agency when we develop learning environments may strengthen and enhance learners’ positioning in relation to their learning. It is our view that though it may not be the ‘purest’ and more humanistic view of agency, by providing a variety of dispositions in learning environments, learners will be given tools that will support them to become more agentic in their learning. We find this important as the HE sector has historically favoured a transmission model of assessment and feedback that position learners as passive receivers of information about their work and not as active agents (Beaumont et al., 2011; Boud & Falchikov, 2006; Nieminen & Tuohilampi, 2020). The lecturer’s role is dominant, leading from the start of the assessment process (writing the assessment brief and the criteria) to the end (grading the work and providing feedback). Turning this dominant role off is not a straightforward endeavour. It is rooted in lecturers’ and students’ identities of how they see their role in higher education.

Winstone, Pitt, and Nash (2021), for example, have looked at understanding how lectures saw their role in assessment and feedback; they compared two models of feedback, one oriented in a cognitivist approach which emphasises the responsibility in the lecturer and another one following a socio-constructivist approach which brings into the foreground responsibilities in the feedback process to the student. In their research, they found that the majority of lecturers were influenced by transmission-based models of feedback with lecturers conveying greater certainty in these types of assessment practices and ‘were more likely to use referents to power and positive emotion, when describing their own as opposed to students’ responsibilities’ (Winstone et al., 2021, p. 118). This dominant role is often ill-defined as it is implicit in centuries of tradition particularly when we discuss assessment. Learners will gain agency in the assessment process if the dominant subject is willing to give away some of that power and if explicit opportunities are provided to the learners to grasp how to develop their active role in the assessment process. We argue in this research that these explicit opportunities may be presented in the DAFS as part of what Charteris and Smardon (2018) refer to as ‘new material agency’.

This study was informed by this notion of power balance. We wanted to understand whether lecturers are willing to let go of some of the power they currently have in the digital assessment and feedback process and how they see these opportunities for promoting agency being developed in the DAFS.

## Methods

Nieminen and Hilppö (2020) argue that learner agency has been under-conceptualised in the context of assessment in HE and that this is leading to methodological approaches that are inconsistent. One of the challenges we had as part of this research was to create a consistent framework for participants to work with when reflecting on existing assessment and feedback practices. As the study participants came from different disciplinary backgrounds and from two different institutions, we wanted to develop a scenario that was common to all. We were informed by participatory design techniques (Casanova et al., 2020; Penuel et al., 2007), in particular using a DAFS mock-up (Additional file 1) and a given scenario (Additional file 2) as a starting point to collect data from discussions about existing practices and to enthuse the creation of new solutions. Participatory processes inject initiative and enthusiasm and lead to collaboration between product designers and users, fostering a sense of shared ownership among these different subjects regarding the end product (Ada, 2018; Casanova et al., 2020). Therefore, researchers are able to gain important insight into the symbiotic relationship between users and the product, which they may not be able to do in more traditional interview settings (ElShaer, Casanova, Freestone & Calabrese,^ 2020^; Schuler & Namioka, 1993).

### Context of the study

This study was developed in two English universities that have been engaged for more than a decade in digital assessment and feedback. Policies have been written to support this wide adoption, lecturers have been trained and supported to create their assessments digitally and there is an expectation that part of the assessment regime will be done digitally. Digital submissions may come in the form of essays, reports, reflections or dissertations depending on the level or discipline of study. Participants (n=58) were selected based on their engagement with the two universities teaching recognition schemes (van der Sluis, Burden, & Huet, 2017); hence, they were perceived as having an interest in pedagogy. Participants were selected from different disciplinary areas and ranged from lecturers to professors. We aimed to have a broad selection of lecturers from different disciplines and experiences to capture the many facets of digital assessment and feedback practices (Twining, Heller, Nussbaum, & Tsai, 2017). Although research has suggested that disciplines influence how online assessment and feedback is perceived and practised (Casanova et al., 2020), we decided that crossing disciplines would provide a diverse range of discussions and a richer set of data.

For the sake of this research, we did not make explicit reference to the type of digital submission as we intended to ensure that all disciplinary practices were covered. There is an expectation in these two institutions that with the digital submission there will be a grade, usually supported by criteria, and feedback, which is either set to justify the grade or to provide recommendations for learners’ improvement in future assessments. All of this process is typically done by a digital assessment tool (Bausili, 2018).

### Data collection

In this study, the data was collected by using ‘sandpits’, which are creative and design-led focus groups (Casanova et al., 2020). These ‘sandpits’ were made up of three parts:

(i) Setting the scene—through storytelling, participants were introduced to the story of the learner and the lecturer when engaging with the DAFSs mock-up. This sets up the scenario to be explored.
(ii) Critique—participants were asked to critique the DAFSs mock-up and its features by creating annotated coloured sticky notes and identifying what they would like to keep (green), change (yellow) or lose (red). This step was important as it allowed for a rich in-depth discussion regarding what they would want to change and how they would carry out this change, as well as fostering a discussion on their assessment and feedback practices.
(iii) Consolidation—participants had some time to propose new features or to redesign some of the existing ones.

Each ‘sandpit’ lasted for approximately 1 h. Ten ‘sandpits’ were carried out in the two institutions.

The researchers were actively involved during the sandpits by setting the scene, reading the scenario and assisting with establishing links with pedagogical and digital practice. The researchers’ role as facilitators was carefully discussed with the participants to clarify possible ethical issues deriving from the researchers’ involvement (Kemmis, McTaggart, & Nixon, 2014).

**Fig. 1:**
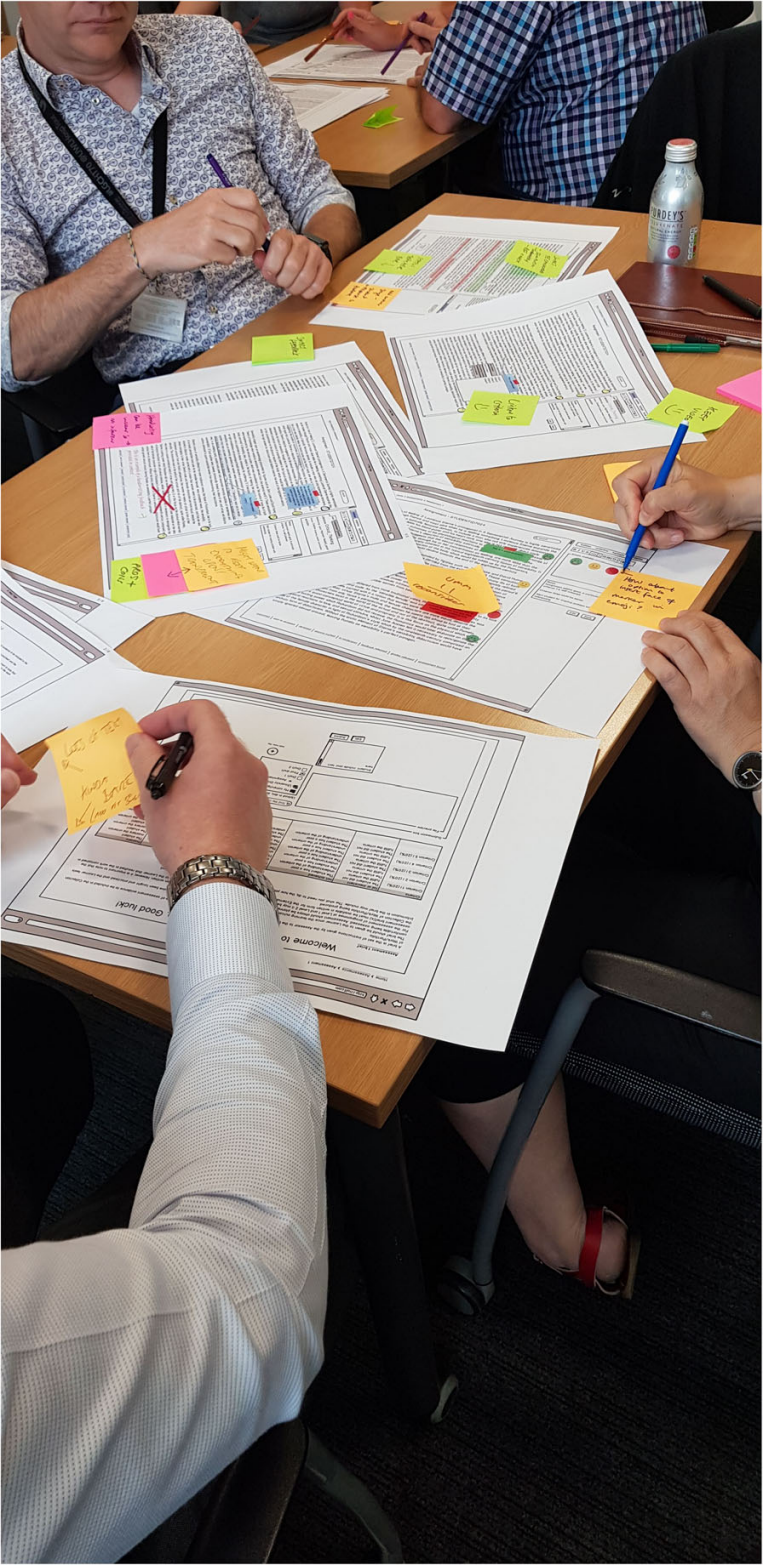
The display of sandpit 1 during stage 2: critique

The conversations were recorded using a sound recorder and then transcribed. Pictures of the sticky notes were also used as secondary datasets to help signpost the design elements of the discussions in each of the ‘sandpits’ (see Fig. 1).

### DAFSs mock-up

The DAFSs mock-up was illustrated using a piece of software called *Balsamic Mockups 3*. By using a mock-up approach, we intended to replicate a participatory design session that is commonly used in software design and to explore the creativity of the participants by asking them to design solutions and suggest new features (Craft, 2013).

The DAFS mock-up was designed to replicate a typical digital assessment and feedback process by incorporating existing practices. However, new and enhanced tools and features, focusing particularly on learner engagement with the feedback, were also introduced.

**Fig. 2:**
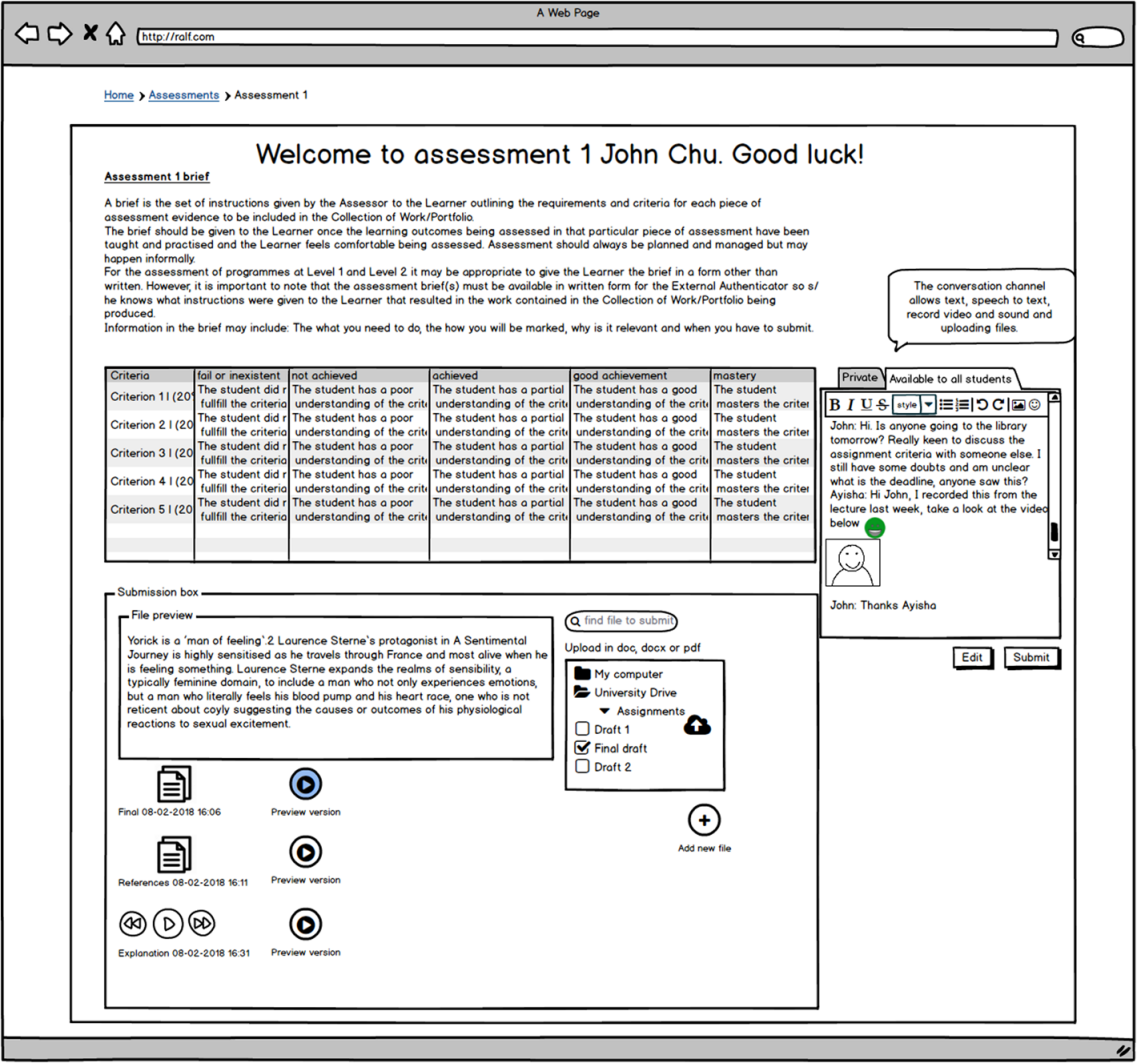
Representation of the screen when John (the learner persona) submits his first assessment attempt

**Fig. 3:**
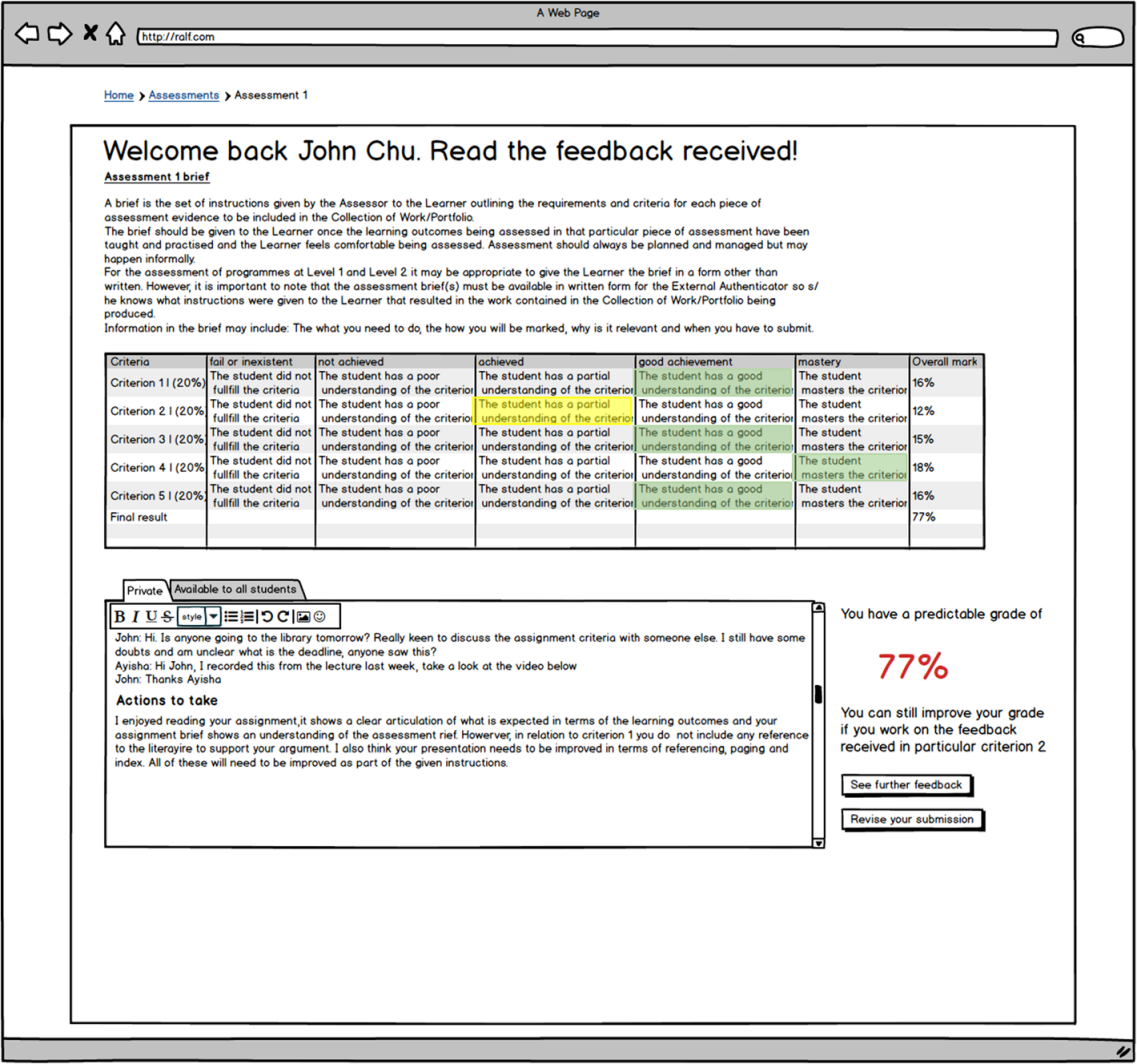
Representation of the screen when John (the learner persona) can resubmit his assignment based on the feedback and actions to take forward

In Figs. 2 and 3, it is possible to see some of the features explored in the DAFS mock-up—in this case, there is an area where learners can ask the lecturer questions (Fig. 2) before submitting their assignment and an area where they can review their feedback and resubmit their assignment (Fig. 3). We illustrated the use of the DAFS using a storytelling technique, which aimed at depicting the mock-up features and hypothetical behaviour of lecturers and learners when using each feature. The storytelling technique was important to ensure that all of the participants were starting the sandpit exercise with the same conceptual framework and to have a wider understanding of what each feature had been designed for. The story comprised of a script read by the researcher and a series of screenshots which presented several stages of the assessment and feedback process which were based on a coursework submission; it is important to mention that although no reference was made to the type of assessment the example illustrated in the mock-up was an essay. We used personas (Earnshaw, Tawfik, & Schmidt, 2017) as a method to provide a detailed description of a fictional user whose characteristics represented a specific user group, in this case, the lecturer and assessor (Ayesha) and the learner (John Chu). Copies of the mock-ups used can be seen in Additional file 1 whilst the detailed description of the scenario is presented in Additional file 2.

### Data analyses

We were informed by the work of scholars like Braun and Clarke (2020) and their perspectives on reflexive thematic analyses (TA) approaches for qualitative research. We agree on the relevance of the researcher’s subjectivity as a resource in the analyses of the data, as part of their conceptions of the theories and how those theories inform the interpretation of the data (Braun & Clarke, 2020). Reflexive TA encourages researchers not to engage in the analytic process as a solely inductive or deductive process but rather to recognise that both can work together in a ‘continuum’ rather than in a ‘dichotomy’ (Clarke & Braun, 2013).

The data was collected and analysed respecting all of the ethical requirements, including anonymity and data confidentiality. As suggested by Braun and Clarke (2020), we started with data familiarisation by reading and re-reading the transcripts from the ‘sandpits’ and initiating data coding using NVIVO12; this enabled us to identify important features of the data that might be relevant to answering the research question. Then, we started with generating the initial themes from coded and collated data (inductive approach). This process resulted in the creation of 18 themes and 127 individual codes. The codes were thought to be entities that captured (at least) one observation of the data (Charmaz, 2006). Although not considered to be a fundamental step in the data validity, as TA draws from the researcher’s subjectivity as a resource in the analysis of the data (Braun & Clarke, 2020), the decision was taken to use the investigator triangulation method with two researchers being involved in the data collection and analysis (Elliott, Fischer, & Rennie, 1999). This decision was taken to ensure consistency in the themes that emerged, and it was done by using a sample set of the data. The themes that emerged were then compared with further data collected from the sandpits, in particular, the written sticky notes which were placed attached to the screenshots of the mock-up during the sandpits (Fig. 3). This allowed us to review the emerged themes and come up with a final 15 themes and 112 codes (Table 1).

**Table 1:** Summary of the data analyses with number of codes per theme

Clusters	Themes	Sandpits	Codes
Preparing for the assessment	*Engagement with the assessment*	*1, 2, 3, 6, 7, and 8*	7
	*Self-assessment*	*1, 2, 6 and 8*	7
	*Discussion before submission*	*2, 7 and 9*	9
	*Engagement with the criteria*	*2, 6 and 8*	7
	*Rubric*	*2, 6 and 9*	5
	*Customisation*	*6 and 8*	2
	*Assessment samples*	*2*	2
	*Group discussions*	*9*	1
	*Peer-feedback*	*2*	2
Formative feedback	*Feedback-loop*	2, 4, 7 and 8	6
	*Feedback storage*	4 and 7	6
	*Formative feedback*	2 and 6	15
	*Non graded*	4	1
Feedback post-submission	*Dispute the grade*	3, 4, 6, 7, 8, 9 and 10	15
	*Engagement with feedback*	3, 4, 6, 7, 8, 9 and 10	17

The cluster creation was supported by the literature about the topic and the researchers’ own reflections about the digital assessment and feedback process. The 15 themes were distributed in the following three clusters of themes: (i) preparation for the assessment, (ii) formative feedback and (iii) feedback post-submission.

## Results and discussions

The first finding from the sandpits was that all of the lecturers found merit in giving away some of the role in the assessment process to the learner. For the sake of this research, this was an important finding, as it has been pointed out how important learner agency is, within the discourse of agency, to have power transmission from the lecturer to the learner (Nieminen & Hilppö, 2020). Without the lecturer giving away some of this power, the learner would struggle to have an agentic role in the assessment:

(…) There is a balance of power. The assessment process seems to be loaded quite often on the part of the tutors… we should be looking at a scenario where you give me some feedback and I challenge you and you challenge me back (participant, sandpit 6).

Although there were clear references to an increased dialogue in the assessment process, an area also addressed by Nicol (2010) and Winstone and Boud (2019), concerns were raised about the time required to monitor learners’ engagement in assessment and feedback. There was a perception that the more lecturers engaged in dialogue with the learner the more time an assessment would take to assess, and this would add to an already busy workload. An example of encouraging student agency was suggested by one participant in sandpit 8 who felt that students should be able to ‘have the possibility of choosing their preferred method of feedback, if they prefer feedback to be verbal, or if they wanted video or audio’.

As they were exploring the different tools and features in the mock-up, almost all of the lecturers involved felt that technology could be used to encourage learner agency. The exception was one lecturer from healthcare, who said that a one-to-one meeting or a group meeting to discuss the feedback would be more relevant and less time-consuming. Perhaps these findings will result in the wider adoption of digital assessment in both institutions and a perception that this may have been the ‘right’ answer to give. One may ask whether if a different method had been used to collect the data, different findings would have been obtained.

Below, we present the three clusters of themes identified during the data collection, and we also discuss how they can be included as part of DAFS design and assessment practices.

### Preparing for the assessment

The mock-ups included some references to ensure engagement with the assessment brief from the outset, for example, a criteria matrix (also called an assessment rubric) and a message box, in which learners could ask the lecturer or their peers questions about the assessment. Increasing learner agency before submission was seen as a critical part of the assessment process. Lecturers felt that mistakes are made by learners due to misinterpretation of the assessment brief or the grading criteria (sandpits 1, 2, 3, 6, 7 and 8).

For example, a DAFS should have a checklist in the assessment brief area where learners are required to respond to a set of questions regarding their understanding of the assessment:

What might be nice is to … include a checklist that could be personalised by the lecturer, not just personalised, but tailored. So for example, you know, make sure you make all your references in this format, make sure that you have included the ethics form, you know, whatever might be additionally needed … just to provide a little bit of scaffolding. (Participant, sandpit 7)

Participants suggested that checklist questions could be related to referencing, similarity, the length of the assessment, their understanding of the assessment brief and the assessment criteria, spell checking or even reflections on the feedback provided in a previous similar assessment (sandpits 2, 6, 7 and 8). A participant, in ‘sandpit’ 6, reinforced this by saying that this approach would be ‘*an intervention to change behaviour’.*

And we liked the idea of the fit to submit box. And we thought that box could maybe be used for the learners to do a self-assessment exercise to check that they had got everything ready. (Participant, sandpit 6)

This would, on the one hand, ensure that learners confirmed themselves whether they had dealt with typical mistakes and, on the other hand, start the submission process earlier. Equally, this could be used as a mean to ensure that learners engage with the assessment criteria before starting to write their submission, which was the main area of concern shared by the participants in sandpit 6.

Other suggestions were also made (i) to provide exemplars of previous assessments to enable learners to improve their understanding of the assessment criteria (sandpit 2), (ii) to self-assess their work against criteria (sandpits 1, 2, 6 and 8), and (iii) to participate in peer-feedback tasks (sandpit 2). These exercises would aim to ensure that learners engaged with the assessment criteria and improve their assessment literacy:

… what I have done in class with my learners is I’ve taken a sample paper from a previous group and then anonymised it and then having them mark it, using the rubric and the assessment brief. Some of them did it okay. Others struggled. But it was starting to make more sense when they could actually have a go at doing it themselves. (Participant, sandpit 2)

The use of exemplars to promote a further clarity of what is intended by the assessment and to allow the learner to engage with this by assessing the piece of work using the assessment criteria and rubrics is a widely discussed practice (Carless & Chan, 2017; Dixon, Hawe, & Hamilton, 2019; Jonsson, 2013).

Using assessment rubrics to aid learners in self or peer-assessing work was supported in four sandpits. All of the participants in these ‘sandpits’ agreed that the use of assessment rubrics allowed learners to compare the assessment against specific criteria and, by doing so, they could engage more actively with the assessment; they had a clear understanding of how they would be assessed and what they were required to do*.* Recent research (Nieminen & Tuohilampi, 2020) highlights the importance of promoting self-assessment especially in summative models of assessment (high-stake), as only at those moments are learners fully engaged with the process.

Whilst supporting and valuing the exercise of engaging with the assessment from the outset, one of the participants was concerned about the feasibility of this approach: *who would monitor the exercise? Why not do it in the classroom?* (participant, sandpit 2)*.* This participant believed that discussing feedback and assessment was more effective when done in a face-to-face environment. This concern was amplified, as it was associated with heavy academic workload pressures and increased time pressure to release grades to learners—the timescale for this ranged from 2 to 3 weeks in both institutions.

As part of this cluster, we recommend that DAFS promote the opportunity for students to self-assess themselves against the grading criteria. To achieve, lecturers will need to publicise and discuss the marking criteria with their learners. That in turn will provide learners with a more in-depth understanding of the assessment brief. DAFS can support this by developing a mechanism that requires the student to self-assess their work before submission, and this task should be monitored by the lecturer to ensure there is a proper engagement with the assessment brief.

### Formative feedback

An area that is widely discussed in the literature is the opportunity for feedback dialogue between the lecturer and the learner. This dialogue is a fundamental step to ensure that learners acquire feedback literacy, which refers to ‘learners’ ability to understand, utilise and benefit from feedback processes’ (Molloy, Boud, & Henderson, 2019, p. 2). Some authors have designed learner-led frameworks for feedback literacy where learners can gradually develop their own competence about acknowledging and acting upon the formative feedback received (Carless & Boud, 2018; Molloy et al., 2019; Winstone et al., 2017). Importantly, this feedback cannot be given only at the end of a sequence of learning, without the time or opportunity for the learner to use it to improve their performance in related tasks (Molloy et al., 2019).

When developing the mock-up, we included different elements to encourage the existence of formative feedback. We deliberately included the ability for learners to comment on each iteration of feedback and ask questions. Equally, as part of the narrative, we included references to the possibility of submitting different versions of the assignment.

Opportunities for formative feedback were perceived as an important step to promote learners’ feedback literacy by the different groups (sandpits 1, 2, 4, 7 and 8). However, concerns were raised about what this should entail. For example, in sandpit 2, it was suggested submitting shorter drafts and the feedback could be used to address some of the assessment criteria as a strategy to avoid the submission of a full draft. Feedback on full draft submissions, although recognised as useful for learners, was thought to be the wrong approach (sandpits 2 and 6), as it might lead to complaints about the inconsistency between the feedback provided on the draft and final versions.

When they show us a draft, again, the University current guidelines are that you just see, like a plan and maybe a sample with a couple of paragraphs, you’re not supposed to see the whole assignment, because that’s seen as giving them an unfair advantage, because you are in effect, marking it but not marking it. And then if they have problems, then it comes back to bite you because they say, but you looked at it, and you didn’t flag anything up. (Participant, sandpit 2)

Furthermore, concerns were raised about the number of iterations on feedback that were allowed in the submission process, as the lecturer could significantly improve the quality of the assessment even if this was not intentional:

When you say that this could be running 4 or 5 times? I guess there is a question about at the end of the day, how much of that work is the learners work? And how much of it is my work? You know … Yes if you’re doing it as part of the formative process, you are writing it for them. (Participant, sandpit 2)

It was also made apparent that there were issues in terms of the time required to do this. Reflecting on this perceived insecurity of lecturers during the assessment process, it is interesting to address the issue of feedback literacy from lecturers’ point of view, as they were not confident about how consistent their feedback would be in the different iterations of the assessment.

To overcome this issue, the suggestion was made for formative feedback to be part of the full learning experience at a course level. This is an interesting finding as currently, this is not an available feature in the majority of DAFS, which tend to be designed at a modular level. This innovation would depend on a programme of study overview of feedback, an area discussed by Boud and Molloy (2013) and Winstone, Nash, Rowntree, and Menezes (2016). The latter argue that modularisation leads to ‘fragmentation’ of the topic covered and disjointed thinking, making modules isolated silos and the feedback on the assessment less able to provide value for future modules.

To address these points, participants suggested that all of the feedback given to learners should be stored somewhere in the DAFS (sandpits 4 and 7); interestingly, this has also been discussed by other authors (Burrows & Shortis, 2011; Parkin et al., 2012; Winstone, 2019). Concerns were raised about the lack of usefulness of the feedback written, and therefore, it was suggested that there should be a mechanism to allow learners to engage with, and action upon the feedback received over time. Feedback would be written and labelled by the lecturer. The labelling system would allow the learner to easily navigate from themes of collected feedback such as referencing, critical thinking or sentence structure. Then, it would be stored in a library of feedback. Learners would then be able to access all of their feedback using metadata and a labelling system. Learners would be able to access the feedback they received before the submission or explore how they could improve the areas of development that had been identified more often as part of the feedback collected during all of their course assignments.

Regarding the possibility of learners being able to extract the feedback received for future assignments or potentially feedback appears on the checklist before learners submit (almost like a nudge). (Participant, sandpit 7)

There was also a suggestion in sandpit 7 that the assessor could check the feedback received by the learners in previous assignments so that they could use this previous feedback to inform their judgement whilst grading. There were also requests for a library of comments to be available for staff to use, allowing them to see past comments made in previous assessments organised by themes and options to appear as they typed (also in sandpit 7). This could also allow for greater consistency whilst grading an area, which some were concerned about.

Creating opportunities for learners to engage with pre-existing feedback was seen as a key area for development for DAFS. The creation of a library of feedback would allow learners to read previous feedback against their current assessment and, potentially, improve areas by signposting to previous mistakes done in earlier submissions. DAFS are typically designed at a module/course level. We recommend that all the feedback is available at a programme level so that both students and lecturers can use it as part of the learning experience.

### Feedback post-submission

Research has suggested that learners are not reading their feedback and are logging into the DAFS only to check their grades (Winstone et al., 2020). This was widely discussed during the sandpits, as lecturers felt pressured to write ‘good’ feedback. Nevertheless, they stated that feedback is often not actioned by the learners (sandpits 3, 4, 6, 7, 8, 9 and 10). We purposely created a button with the title ‘agree with feedback’, which learners would need to press to confirm their grade. This element was the most controversial topic of discussion, as lecturers felt that the system was giving too many powers to the learner. The power balance was therefore questioned at this point.

And also, we felt where it says ‘agree with feedback’ that was a bit controversial… So could it have something that would require somebody to accept the feedback after they’ve read it, because this indicates when you go in straight away, the learner gets their mark, that as we know, learners aren’t reading feedback. So to encourage that, could we have something where they have to see their feedback first go through, tick a box to say they’ve actually read it? And then they get access to their mark following that? (Participant, sandpit 3).

So, coming back to the mark agreed or not agreed if the mark wasn’t valid until the learners have read the feedback that would be a very powerful way to ensure that learners do read the feedback. (Participant, sandpit 4).

You could make that part of the, you know, it’s getting them to interact with feedforward, basically, and getting some form of acknowledgement from them that they understand what you’re talking about. … At some point, they would need to write or reflect on the feedback before the mark is released … But it is not just pressing the button to say that they have read it but there has to be some form of proof that they did, in fact, engage with the feedback (Participant, sandpit 8)

Participants agreed with the need for the learner to act and engage with the end-process, but they did not like the requirement for learners to agree with the feedback, as this would create tensions between the learner and the lecturer (sandpits 3, 4, 6, 7, 8, 9 and 10). There was a feeling among participants that learners frequently confused feedback with the grade and, thus, when considering the feedback received, they were arguing about the grade. This is an argument embedded into the marketisation culture of HE, which is particularly prevalent across some countries, where learners pay high tuition fees and therefore feel empowered, often seeing themselves as consumers (Woodall, Hiller, & Resnick, 2014). Nevertheless, participants did see some value in promoting discussions about the feedback, particularly if learners had to act upon the feedback before the release of their grade, for example, by reflecting on changes they would need to make for future similar assignments (sandpits 4 and 8), and/or providing a rationale for not agreeing with the feedback (sandpits 4, 9 and 10). This strategy has been discussed in the literature by Nicol (2007) and Parkin et al. (2012), who found that learners are more likely to engage with the process of reflection when they have been told explicitly that they will be required to reflect on their feedback before receiving their grade. This ensures that learners engage more actively with the feedback by trying to make sense of it and creating their pathway for development.

Although the majority of the lecturers involved in the sandpits were encouraged by a solution of encouraging learners to engage with the feedback before the release of the grade, this is something that DAFS does not have built-in at present, and it is difficult to replicate pedagogically. DAFS have been typically developed to allow grades and feedback to be released simultaneously; there is no separation between these two elements of assessment. The consequence is that often learners do not read the feedback as they rather prefer to concentrate on the grade which is what they value. To ensure learners use and engage with the feedback, we recommend that the feedback should be sent to the learner before the grade, i.e. to be seen as a separate element. Only after the student reads and engages with the feedback (through reflection or setting up an action plan for improvement in the future) should the grade be released to the student. This will ensure that the feedback written by the lecturer is well understood by the students and eventually acted upon for future assessments. It will encourage a new and more holist culture of assessment which suggests that students also have an active role in the quality of the feedback process.

## Conclusions

In this study, we aimed at providing an answer to the question: how can lecturers and universities improve digital assessment and feedback systems (DAFS) that lead to an increase in learner agency in assessment and feedback? We started by providing evidence that lecturers are open to giving away some of their existing powers in the assessment process. That is of paramount importance to ensure the balance of agency in assessment and feedback.

In this research, we were informed by the work from Charteris and Smardon (2018) and their view of ‘new material agency’, whereby incorporating something new in the learning process may result in different demands on learners, who in turn may develop different competencies. The mock-up used in the sandpits aimed at encouraging lecturers to find new features and tools that would encourage learners to engage differently in the assessment and feedback process. As we found from the data analyses, lecturers were willing to use the DAFS as a mean to find strategies and tools to increase learner agency in the assessment and feedback process. Examples of strategies and tools that emerged from the sandpits were distributed in three main clusters of themes: (i) preparation for the assessment, (ii) formative feedback and (iii) feedback post-submission. It is important to note that the majority of these themes are discussed widely in the literature, and some are already existing practices, so this research is not contributing to the discovery of new knowledge on how to encourage further agency in the assessment and feedback process per se. However, what this research is presenting is how that agency can be exercised in DAFS, in particular how academic staff envisage this process to be taken in digital assessment and feedback. This may be of importance for academic staff, HE institutions and DAFS developers.

Based on participants’ comments during the sandpits, we believe that by changing some features of how DAFS are designed we can contribute to improving digital assessment and feedback and increase learner agency. Throughout the paper, we recommend three areas learner agency can be promoted in DAFS: (i) to promote self-assessment exercises before submission, (ii) to enable a programme-level library of feedback and (iii) to set-up a mechanism whereby grades are only sent to the learner after the feedback is acted upon.

The data for this study were collected in the UK between 2019 and 2020. One limitation of this study is the context where this research was done, which may suggest an overgeneralisation of the findings. Assessment practices in the UK may be different from other countries, and this needs to be taken into consideration when reading the recommendations. Equally, we agree with Twining et al. (2017) that samples should be broad enough to capture the many facets of the phenomenon being researched. It is now our aim to involve learners in discussions around, and critiquing of the same mock-up, using the same method for data collection. We aim to compare and contrast the findings of these two subjects. We are particularly interested in investigating how willing learners are to participate more actively in the assessment and feedback process when offered that possibility and in exploring how they would engage with the recommendations identified during the lecturers’ sandpits. We are also keen to explore this research in other countries particularly those with a different culture of learning and teaching to that of the UK.

## Supplementary Information

**Additional file 1.** DAFS mock-up.

**Additional file 2.** Agency feedback–scenario 2.

## Data Availability

Due to current data protection legislation, the data used for this study cannot be made publicly available. However, some research materials can be made available upon request.

## Abbreviations

EMA: Electronic management of assessment
FEATS: Feedback Engagement and Tracking System
HE: Higher education
SMI: SuperMarkIt
TA: Thematic analyses
