# Supplementary material for: Giving away some of their powers! Towards learner agency in digital assessment and feedback
Source: Res Pract Technol Enhanc Learn. 2021 Jul 7;16(1):20. doi: 10.1186/s41039-021-00168-6 (PMC8261811; doi:10.1186/s41039-021-00168-6)
Supplement: Supplementary file 1 — Additional file 1. DAFS mock-up. [file 41039_2021_168_MOESM1_ESM.pdf]

A Web Page

http://ralf.com

Home > Assessments > Assessment 1

Welcome to assessment 1 John Chu. Good luck!

Assessment 1 brief

A brief is the set of instructions given by the Assessor to the Learner outlining the requirements and criteria for each piece of assessment evidence to be included in the Collection of Work/Portfolio.
The brief should be given to the Learner once the learning outcomes being assessed in that particular piece of assessment have been taught and practised and the Learner feels comfortable being assessed. Assessment should always be planned and managed but may happen informally.
For the assessment of programmes at Level 1 and Level 2 it may be appropriate to give the Learner the brief in a form other than written. However, it is important to note that the assessment brief(s) must be available in written form for the External Authenticator so s/he knows what instructions were given to the Learner that resulted in the work contained in the Collection of Work/Portfolio being produced.
Information in the brief may include: The what you need to do, the how you will be marked, why is it relevant and when you have to submit.

The conversation channel allows text, speech to text, record video and sound and uploading files.

Criteria	fail or inexistent	not achieved	achieved	good achievement	mastery
Criterion 1 I (20	The student did r fullfill the criteria	The student has a poor understanding of the crit	The student has a partial understanding of the crit	The student has a good understanding of the crit	The student masters the criter
Criterion 2 I (20	The student did r fullfill the criteria	The student has a poor understanding of the crit	The student has a partial understanding of the crit	The student has a good understanding of the crit	The student masters the criter
Criterion 3 I (20	The student did r fullfill the criteria	The student has a poor understanding of the crit	The student has a partial understanding of the crit	The student has a good understanding of the crit	The student masters the criter
Criterion 4 I (20	The student did r fullfill the criteria	The student has a poor understanding of the crit	The student has a partial understanding of the crit	The student has a good understanding of the crit	The student masters the criter
Criterion 5 I (20	The student did r fullfill the criteria	The student has a poor understanding of the crit	The student has a partial understanding of the crit	The student has a good understanding of the crit	The student masters the criter

Private | Available to all students

B I U S style

John: Hi. Is anyone going to the library tomorrow? Really keen to discuss the assignment criteria with someone else. I still have some doubts and am unclear what is the deadline, anyone saw this?
Ayisha: Hi John, I recorded this from the lecture last week, take a look at the video below

John: Thanks Ayisha

Edit | Submit

Submission box

File preview

find file to submit

Upload in doc, docx or pdf

- My computer
- University Drive
  - Assignments
- [ ] Draft 1
- [x] Final draft
- [ ] Draft 2

Add new file

A Web Page

http://ralf.com

Home > Assessments > Assessment 1

Welcome to assessment 1 John Chu. Good luck!

Assessment 1 brief

A brief is the set of instructions given by the Assessor to the Learner outlining the requirements and criteria for each piece of assessment evidence to be included in the Collection of Work/Portfolio.
The brief should be given to the Learner once the learning outcomes being assessed in that particular piece of assessment have been taught and practised and the Learner feels comfortable being assessed. Assessment should always be planned and managed but may happen informally.
For the assessment of programmes at Level 1 and Level 2 it may be appropriate to give the Learner the brief in a form other than written. However, it is important to note that the assessment brief(s) must be available in written form for the External Authenticator so s/he knows what instructions were given to the Learner that resulted in the work contained in the Collection of Work/Portfolio being produced.
Information in the brief may include: The what you need to do, the how you will be marked, why is it relevant and when you have to submit.

The conversation channel allows text, speech to text, record video and sound and uploading files.

Criteria	fail or inexistent	not achieved	achieved	good achievement	mastery
Criterion 1 I (20	The student did r fullfill the criteria	The student has a poor understanding of the crit	The student has a partial understanding of the crit	The student has a good understanding of the crit	The student masters the criter
Criterion 2 I (20	The student did r fullfill the criteria	The student has a poor understanding of the crit	The student has a partial understanding of the crit	The student has a good understanding of the crit	The student masters the criter
Criterion 3 I (20	The student did r fullfill the criteria	The student has a poor understanding of the crit	The student has a partial understanding of the crit	The student has a good understanding of the crit	The student masters the criter
Criterion 4 I (20	The student did r fullfill the criteria	The student has a poor understanding of the crit	The student has a partial understanding of the crit	The student has a good understanding of the crit	The student masters the criter
Criterion 5 I (20	The student did r fullfill the criteria	The student has a poor understanding of the crit	The student has a partial understanding of the crit	The student has a good understanding of the crit	The student masters the criter

Private | Available to all students

John: Hi. Is anyone going to the library tomorrow? Really keen to discuss the assignment criteria with someone else. I still have some doubts and am unclear what is the deadline, anyone saw this?
Ayisha: Hi John, I recorded this from the lecture last week, take a look at the video below

John: Thanks Ayisha

Edit | Submit

Submission box

File preview

Yorick is a 'man of feeling'.2 Laurence Sterne's protagonist in A Sentimental Journey is highly sensitised as he travels through France and most alive when he is feeling something. Laurence Sterne expands the realms of sensibility, a typically feminine domain, to include a man who not only experiences emotions, but a man who literally feels his blood pump and his heart race, one who is not reticent about coyly suggesting the causes or outcomes of his physiological reactions to sexual excitement.

find file to submit

Upload in doc, docx or pdf

- My computer
- University Drive
  - Assignments
- Draft 1
- Final draft
- Draft 2

Add new file

Final 08-02-2018 16:06 — Preview version

References 08-02-2018 16:11 — Preview version

Explanation 08-02-2018 16:31 — Preview version

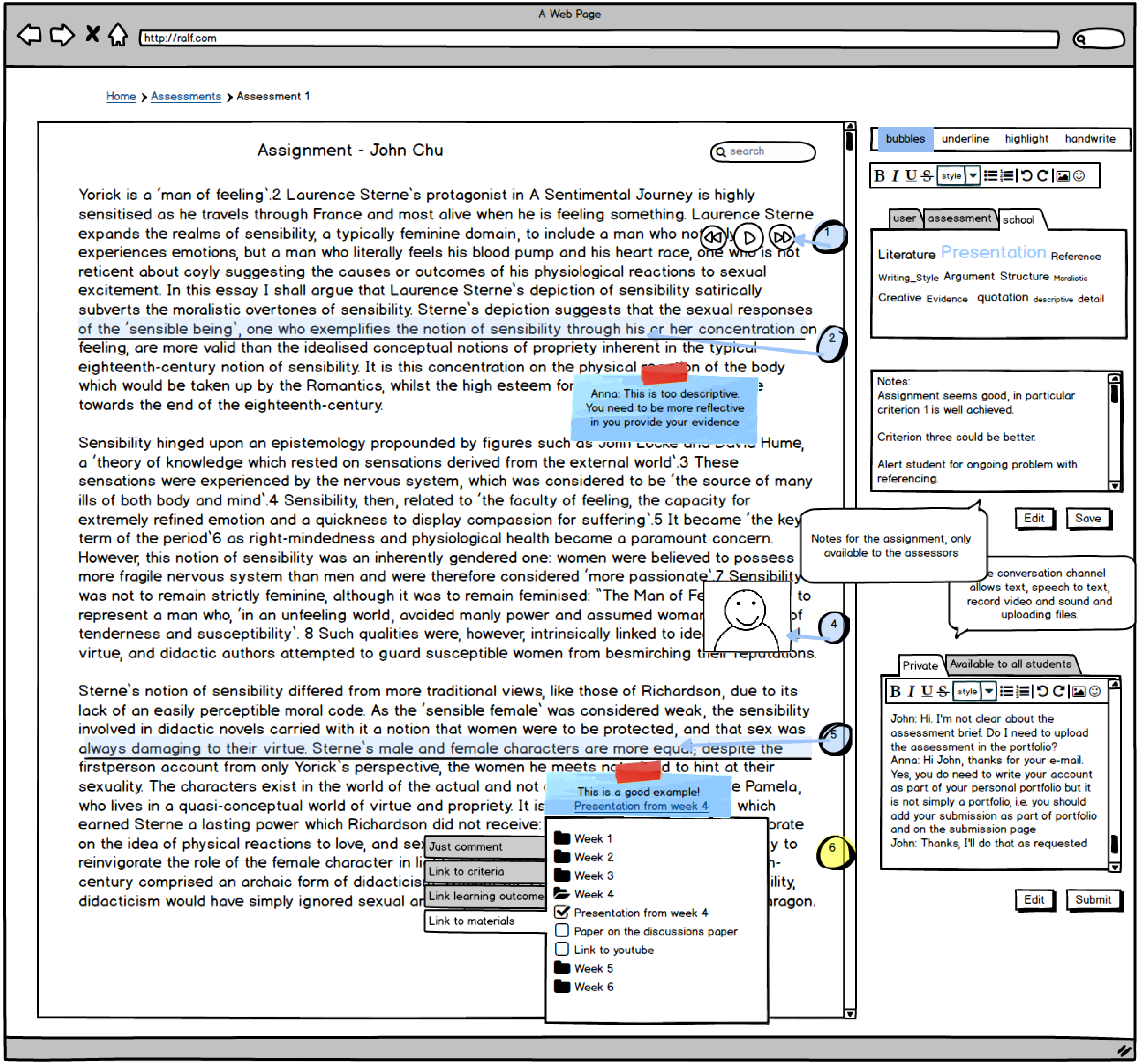
A Web Page
http://ralf.com
Home > Assessments > Assessment 1
Assignment - John Chu
search
Yorick is a 'man of feeling'.2 Laurence Sterne's protagonist in A Sentimental Journey is highly sensitised as he travels through France and most alive when he is feeling something. Laurence Sterne expands the realms of sensibility, a typically feminine domain, to include a man who no
experiences emotions, but a man who literally feels his blood pump and his heart race, one who is not reticent about coyly suggesting the causes or outcomes of his physiological reactions to sexual excitement. In this essay I shall argue that Laurence Sterne's depiction of sensibility satirically subverts the moralistic overtones of sensibility. Sterne's depiction suggests that the sexual responses of the 'sensible being', one who exemplifies the notion of sensibility through his or her concentration on feeling, are more valid than the idealised conceptual notions of propriety inherent in the typical eighteenth-century notion of sensibility. It is this concentration on the physical
n of the body which would be taken up by the Romantics, whilst the high esteem for
towards the end of the eighteenth-century.
Anna: This is too descriptive. You need to be more reflective in you provide your evidence
Sensibility hinged upon an epistemology propounded by figures such
Hume, a 'theory of knowledge which rested on sensations derived from the external world'.3 These sensations were experienced by the nervous system, which was considered to be 'the source of many ills of both body and mind'.4 Sensibility, then, related to 'the faculty of feeling, the capacity for extremely refined emotion and a quickness to display compassion for suffering'.5 It became 'the key term of the period'6 as right-mindedness and physiological health became a paramount concern. However, this notion of sensibility was an inherently gendered one: women were believed to possess more fragile nervous system than men and were therefore considered 'more passionate'.7 Sensibility was not to remain strictly feminine, although it was to remain feminised: "The Man of Fe
to represent a man who, 'in an unfeeling world, avoided manly power and assumed woman
of tenderness and susceptibility'. 8 Such qualities were, however, intrinsically linked to ide
virtue, and didactic authors attempted to guard susceptible women from besmirching
Sterne's notion of sensibility differed from more traditional views, like those of Richardson, due to its lack of an easily perceptible moral code. As the 'sensible female' was considered weak, the sensibility involved in didactic novels carried with it a notion that women were to be protected, and that sex was always damaging to their virtue. Sterne's male and female characters are more equ
despite the firstperson account from only Yorick's perspective, the women he meets n
d to hint at their sexuality. The characters exist in the world of the actual and not
e Pamela, who lives in a quasi-conceptual world of virtue and propriety. It is
which earned Sterne a lasting power which Richardson did not receive
orate on the idea of physical reactions to love, and se
y to reinvigorate the role of the female character in li
century comprised an archaic form of didacticis
ility, didacticism would have simply ignored sexual a
ragon.
This is a good example!
Presentation from week 4
Just comment
Link to criteria
Link learning outcome
Link to materials
Week 1
Week 2
Week 3
Week 4
Presentation from week 4
Paper on the discussions paper
Link to youtube
Week 5
Week 6
1
2
4
5
6
bubbles
underline
highlight
handwrite
B I U S style
user
assessment
school
Literature Presentation Reference
Writing_Style Argument Structure Moralistic
Creative Evidence quotation descriptive detail
Notes:
Assignment seems good, in particular criterion 1 is well achieved.
Criterion three could be better.
Alert student for ongoing problem with referencing.
Edit
Save
Notes for the assignment, only available to the assessors
e conversation channel allows text, speech to text, record video and sound and uploading files.
Private
Available to all students
B I U S style
John: Hi. I'm not clear about the assessment brief. Do I need to upload the assessment in the portfolio?
Anna: Hi John, thanks for your e-mail. Yes, you do need to write your account as part of your personal portfolio but it is not simply a portfolio, i.e. you should add your submission as part of portfolio and on the submission page
John: Thanks, I'll do that as requested
Edit
Submit

A Web Page

http://ralf.com

Home > Assessments > Assessment 1

Welcome back John Chu. Read the feedback received!

Assessment 1 brief

A brief is the set of instructions given by the Assessor to the Learner outlining the requirements and criteria for each piece of assessment evidence to be included in the Collection of Work/Portfolio.
The brief should be given to the Learner once the learning outcomes being assessed in that particular piece of assessment have been taught and practised and the Learner feels comfortable being assessed. Assessment should always be planned and managed but may happen informally.
For the assessment of programmes at Level 1 and Level 2 it may be appropriate to give the Learner the brief in a form other than written. However, it is important to note that the assessment brief(s) must be available in written form for the External Authenticator so s/he knows what instructions were given to the Learner that resulted in the work contained in the Collection of Work/Portfolio being produced.
Information in the brief may include: The what you need to do, the how you will be marked, why is it relevant and when you have to submit.

Criteria	fail or inexistent	not achieved	achieved	good achievement	mastery	Overall mark
Criterion 1 I (20%)	The student did not fullfill the criteria	The student has a poor understanding of the criterion	The student has a partial understanding of the criterion	The student has a good understanding of the criterion	The student masters the criterion	16%
Criterion 2 I (20%)	The student did not fullfill the criteria	The student has a poor understanding of the criterion	The student has a partial understanding of the criterion	The student has a good understanding of the criterion	The student masters the criterion	12%
Criterion 3 I (20%)	The student did not fullfill the criteria	The student has a poor understanding of the criterion	The student has a partial understanding of the criterion	The student has a good understanding of the criterion	The student masters the criterion	15%
Criterion 4 I (20%)	The student did not fullfill the criteria	The student has a poor understanding of the criterion	The student has a partial understanding of the criterion	The student has a good understanding of the criterion	The student masters the criterion	18%
Criterion 5 I (20%)	The student did not fullfill the criteria	The student has a poor understanding of the criterion	The student has a partial understanding of the criterion	The student has a good understanding of the criterion	The student masters the criterion	16%
Final result						77%

Private | Available to all students

B I U S style

John: Hi. Is anyone going to the library tomorrow? Really keen to discuss the assignment criteria with someone else. I still have some doubts and am unclear what is the deadline, anyone saw this?
Ayisha: Hi John, I recorded this from the lecture last week, take a look at the video below
John: Thanks Ayisha

Actions to take

I enjoyed reading your assignment,it shows a clear articulation of what is expected in terms of the learning outcomes and your assignment brief shows an understanding of the assessment rief. However, in relation to criterion 1 you do not include any reference to the literayire to support your argument. I also think your presentation needs to be improved in terms of referencing, paging and index. All of these will need to be improved as part of the given instructions.

You have a predictable grade of

77%

You can still improve your grade if you work on the feedback received in particular criterion 2

See further feedback

Revise your submission

A Web Page

http://ralf.com

[Home](#) > [Assessments](#) > Assessment 1

Assignment - John Chu

search

Yorick is a 'man of feeling'.2 Laurence Sterne's protagonist in A Sentimental Journey is highly sensitised as he travels through France and most alive when he is feeling something. Laurence Sterne expands the realms of sensibility, a typically feminine domain, to include a man who no[illegible] experiences emotions, but a man who literally feels his blood pump and his heart race, one who is not reticent about coyly suggesting the causes or outcomes of his physiological reactions to sexual excitement. In this essay I shall argue that Laurence Sterne's depiction of sensibility satirically subverts the moralistic overtones of sensibility. Sterne's depiction suggests that the sexual responses of the 'sensible being', one who exemplifies the notion of sensibility through his or her concentration on feeling, are more valid than the idealised conceptual notions of propriety inherent in the typical eighteenth-century notion of sensibility. It is this concentration on the physical [illegible]on of the body which would be taken up by the Romantics, whilst the high esteem for [illegible] towards the end of the eighteenth-century.

Anna: This is too descriptive. You need to be more reflective in you provide your evidence

John: Hi! Can you provide further explanations please?

Sensibility hinged upon an epistemology propounded by figures such [illegible] Hume, a 'theory of knowledge which rested on sensations derived from the [illegible] sensations were experienced by the nervous system, which was con[illegible] of many ills of both body and mind'.4 Sensibility, then, related to 'the faculty of feeling, the capacity for extremely refined emotion and a quickness to display compassion for suffering'.5 It became 'the key term of the period'6 as right-mindedness and physiological health became a paramount concern. However, this notion of sensibility was an inherently gendered one: women were believed to possess a more fragile nervous system than men and were therefore considered 'more passionate'.7 Sensibility was not to remain strictly feminine, although it was to remain feminised: "The Man of Fe[illegible] to represent a man who, 'in an unfeeling world, avoided manly power and assumed woman[illegible] of tenderness and susceptibility'. 8 Such qualities were, however, intrinsically linked to ide[illegible]d virtue, and didactic authors attempted to guard susceptible women from besmirching their reputations.

Sterne's notion of sensibility differed from more traditional views, like those of Richardson, due to its lack of an easily perceptible moral code. As the 'sensible female' was considered weak, the sensibility involved in didactic novels carried with it a notion that women were to be protected, and that sex was always damaging to their virtue. Sterne's male and female characters are more equal; despite the firstperson account from only Yorick's perspective, the women he meets no[illegible]d to hint at their sexuality. The characters exist in the world of the actual and not [illegible]e Pamela, who lives in a quasi-conceptual world of virtue and propriety. It is [illegible] which earned Sterne a lasting power which Richardson did not receive: [illegible] elaborate on the idea of physical reactions to love, and sexual stimulus, whil[illegible]essary to reinvigorate the role of the female character in light of Richardso[illegible]teenth-century comprised an archaic form of didacticism. Without the erotic [illegible] sensibility, didacticism would have simply ignored sexual arousal, leaving Pamela as sensibility's perfect paragon.

This is a good example! [Presentation from week 4](#)

John: Ok. I'll change that accordingly. I think I know what I need to do now :)

1 2 3 4 5 6

bubbles | underline | highlight | handwrite

The conversation channel allows text, speech to text, record video and sound and uploading files.

Private | Available to all students

John: Hi. I'm not clear about the assessment brief. Do I need to upload the assessment in the portfolio?
Anna: Hi John, thanks for your e-mail. Yes, you do need to write your account as part of your personal portfolio but it is not simply a portfolio, i.e. you should add your submission as part of portfolio and on the submission page
John: Thanks, I'll do that as requested

The student can use the public assessment channel to discuss with colleagues the way forward, schedule a meeting or discuss the feedback received.

Edit | Submit

A Web Page

http://ralf.com

Home > Assessments > Assessment 1

Welcome to assessment 1 John Chu. Good luck!

Assessment 1 brief

A brief is the set of instructions given by the Assessor to the Learner outlining the requirements and criteria for each piece of assessment evidence to be included in the Collection of Work/Portfolio.
The brief should be given to the Learner once the learning outcomes being assessed in that particular piece of assessment have been taught and practised and the Learner feels comfortable being assessed. Assessment should always be planned and managed but may happen informally.
For the assessment of programmes at Level 1 and Level 2 it may be appropriate to give the Learner the brief in a form other than written. However, it is important to note that the assessment brief(s) must be available in written form for the External Authenticator so s/he knows what instructions were given to the Learner that resulted in the work contained in the Collection of Work/Portfolio being produced.
Information in the brief may include: The what you need to do, the how you will be marked, why is it relevant and when you have to submit.

Criteria	fail or inexistent	not achieved	achieved	good achievement	mastery	Overall mark
Criterion 1 I (20%)	The student did not fullfill the criteria	The student has a poor understanding of the criterio	The student has a partial understanding of the criterio	The student has a good understanding of the criterio	The student masters the criterio	16%
Criterion 2 I (20%	The student did not fullfill the criteria	The student has a poor understanding of the criterio	The student has a partial understanding of the criterio	The student has a good understanding of the criterio	The student masters the criterio	17%
Criterion 3 I (20%	The student did not fullfill the criteria	The student has a poor understanding of the criterio	The student has a partial understanding of the criterio	The student has a good understanding of the criterio	The student masters the criterio	15%
Criterion 4 I (20%	The student did not fullfill the criteria	The student has a poor understanding of the criterio	The student has a partial understanding of the criterio	The student has a good understanding of the criterio	The student masters the criterio	18%
Criterion 5 I (20%	The student did not fullfill the criteria	The student has a poor understanding of the criterio	The student has a partial understanding of the criterio	The student has a good understanding of the criterio	The student masters the criterio	15%
Final result						81%

Private | Available to all students

B I U S style

Actions to take

I enjoyed reading your assignment, it shows a clear articulation of what is expected in terms of the learning outcomes and your assignment brief shows an understanding of the assessment rief. However, in relation to criterion 1 you do not include any reference to the literayire to support your argument. I also think your presentation needs to be improved in terms of referencing, paging and index. All of these will need to be improved as part of the given instructions.

Overall result

I enjoyed reading your assignment in particular how you were able to incorporate the different learning outcomes in your assessment.

- Good grasp of learning outcomes
- Alignment with the assessment brief
- Well articulated text with sound evidence of use of references to support argument

Feedforward

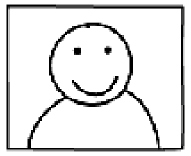

You have a

81%

Which is a good achievement!

Read all your feedback
