# Supplementary material for: Giving away some of their powers! Towards learner agency in digital assessment and feedback
Source: Res Pract Technol Enhanc Learn. 2021 Jul 7;16(1):20. doi: 10.1186/s41039-021-00168-6 (PMC8261811; doi:10.1186/s41039-021-00168-6)
Supplement: Supplementary file 2 — Additional file 2. Agency feedback–scenario 2. [file 41039_2021_168_MOESM2_ESM.pdf]

Agency in feedback – scenario 2

Scenario 2 introduces the concept of agency emphasising the role of the student in managing their feedback

1- **Student** John Chu’s is the student name. John is a literature student at the University. John needs to submit an essay in the Submit-it platform. John can see the assessment page with the corresponding assessment brief and the criteria presented in a form of a rubric.
   In this scenario, both the assessor and the student are not anonymous (i.e. student name is available both in the top of the page and student name and assessor name are made available in the comment box). On the right side there is a conversation channel box feature for the specific assessment. John can use it to ask questions to his teacher or to his fellow students before submission if he has any doubts, during the submission to alert for a specific issue with the submission or after the feedback is received. There are two options: (a) one by default private only available to the student and the assessor; (b) another one public and available to all students involved in the assessment and the assessors. The conversation channel allows text, speech to text, emojis, record video and sound and uploading files.

2- **Student** John submits his essay for the first time as a word document. John has the possibility of accompanying this submission with additional files. The submitted file becomes a live document in the system and will be edited/changed without future uploads, as part of further iterations between John and Ayisha, the assessor. John can add other type of files, such as video or sound to provide further explanations or evidences.

3- **Assessor** The assignment with the received feedback is now presented. In the first section, a search box is available allowing Ayisha to find any word easily. In the assignment window there are a variety of options displayed as in-text feedback. Sound feedback, underlining with comments, video and comments with a link to materials. All of this are signposted by a balloon icon with a number (yellow=disabled; blue=enabled). On the right navigation side, Ayisha has a variety of options to provide feedback

   a. Ayisha can use provide in-text feedback in two ways, either following the text editor (with the options bubbles, underline, highlight and handwriting) or following the option quick mark (with the options library from the user, library from the specific assessment or library from the school). After inserting their comment, they can signpost each comment to specific criterion, the learning outcome or a specific learning material available in the VLE. In-text video/podcast snippets/screencast are also available options.
   b. A comment box is available for notetaking albeit only available to the assessors' team and to the external examiner.
   c. The conversation channel with the option of private comments or comments available to all students. In this box Ayisha can provide general feedback to John using the private option.

4- **Student** John receives a notification in is phone that a new grade and feedback are available in Submit-it.

   a. He accesses the assignment via the assignment page where his able to see the rubric highlighted in the mark achieved for each

criterion (red, yellow and green). A criterion mark and predictable overall mark is also available – John got a 77%.

b. John receives a general feedback comment below the rubric with the heading Actions to take.
c. On the right-end side John can read a message with the text “you can still improve your grade if you work on the feedback received, in particular to respond to criterion 2”. Below the message there are two options are available: ‘see further feedback’ or ‘revise your submission’

5- **Student** On the feedback page John can respond to the comments by clicking on the balloon text. He can only use the in-text editor to respond to the assessor or to edit the text in the live document. For more generic comments John can use the conversation channel box with the private option enabled. After finishing addressing all the actions to take John re-submits his assessment. The process is iterative and can take up to 5 times.

6- **Student** In the final stage Ayisha provides general feedback using a marking rubric and use this rubric as a marking schema to provide the final grade. John receives a notification in is phone that a new grade and feedback are available in Submit-it. He can see the final grade, the rubric displayed and the private feedback.

a. In the private assessment conversation channel the assessor provides two iterations of feedback
    i. A general comment providing feedback in bullet points summarising the rationale for the grade
    ii. A feedforward section with a video highlighting where John needs to improve for further assessments.

John can still use the private assessment conversation channel to comment the feedback received, including questions and doubts

Question to guide discussions

- Do you prefer feedback to be anonymous or to be able to engage with the assessor using your and her name+?
- Do you like the idea of having a possibility to improve your assignment even if the mark is above the passing mark? For how many times would you be willing to be involved in this process?
- Is the rubric enough to provide feedback? Does this seem more transparent?
- Do you like the idea of ‘actions to take’, ‘overall result’ and ‘feedforward’ to signpost the type of feedback? Do you like feedback to be provided in video?
